# FDA 2025 Cancer Drug Approvals: targeted therapy dominates

**DOI:** 10.37349/etat.2026.1002369

**Published:** 2026-04-27

**Authors:** Jan Trøst Jørgensen

**Affiliations:** IRCCS Azienda Ospedaliero-Universitaria di Bologna, Italy; Medical Sciences, Dx-Rx Institute, 3480 Fredensborg, Denmark

**Keywords:** FDA, drug approvals, oncology, hematology, precision medicine, biomarkers, companion diagnostics, drug development

## Abstract

This commentary discusses the FDA’s drug approvals in 2025, with a particular focus on cancer therapies and the role of companion diagnostics (CDx). Cancer has emerged as the leading therapeutic area, accounting for 35% of all new drug approvals, largely driven by targeted therapies, with kinase inhibitors representing nearly half of these drugs. Many of the drugs have received orphan drug designations and/or have utilized the Accelerated Approval Program. A key finding was the widespread adoption of the drug-diagnostic co-development model, in which a CDx assay is developed along with the drug and used for patient selection in clinical trials. However, a significant challenge is the frequent lack of concurrent drug and CDx assay approvals. The absence of an analytically and clinically validated CDx assay may pose a challenge for healthcare providers in accurately identifying eligible patients, potentially delaying access to appropriate therapy. The FDA’s cancer drug approvals for 2025 highlight an ongoing commitment to precision medicine, with several new targeted treatments, such as antibody-drug conjugates and kinase inhibitors, where CDx assays play an important role in identifying the appropriate patient population.

## Introduction

The number of targeted cancer drugs has increased considerably in recent years, and many have been developed using the drug diagnostic codevelopment model. In this model, a predictive biomarker assay is developed in parallel with the drug to identify likely responding patients. The development of these assays is based on our increased understanding of tumor biology and drug mechanisms of action. Regulators have classified these predictive biomarker assays as companion diagnostics (CDx). The FDA defines a CDx assay as an in vitro diagnostic device or an imaging tool that provides information that is essential for the safe and effective use of a corresponding therapeutic product [1]. The first time a CDx assay was developed concomitantly with a targeted cancer drug was in the 1990s, when the monoclonal antibody trastuzumab was developed for HER2-overexpressing metastatic breast cancer [2]. An immunohistochemical (IHC) assay was developed along with the drug to identify HER2 tumor expression, aiming to select patients who might respond to treatment. In 1998, both trastuzumab and the IHC assay HercepTest were approved by the FDA [3]. This concurrent approval was important because it ensured the availability of a CDx assay that was both analytically and clinically validated, thereby facilitating accurate patient selection for treatment. In breast cancer, approximately 20% of patients overexpress the HER2 protein or have *HER2* amplification and are hence candidates for treatment with trastuzumab [4].

The development that started with trastuzumab and HercepTest more than 25 years ago has continued, and by the end of 2025, the number of targeted cancer drugs associated with a CDx assay has increased to more than 80 [5]. Reviewing the 2025 FDA approvals for drugs indicated for the treatment of different oncological and hematological malignancies, this development seems to be continuing at an increased pace [6]. This brief commentary examines the cancer drugs that received FDA approval in 2025, with a particular focus on those developed using the drug-diagnostic co-development approach. Based on data available in the database for FDA-approved drugs (Drugs@FDA), the clinical efficacy documentation that led to their approval was reviewed, with specific attention given to predictive biomarkers, clinical trial design, and recommendations for patient selection [7]. Information regarding orphan drug status, accelerated approval designation, and regulatory-approved CDx assays was obtained from the following FDA databases: “Search Orphan Drug Designations and Approvals,” “Drugs@FDA,” and “List of Cleared or Approved Companion Diagnostic Devices (In Vitro and Imaging Tools),” respectively [5, 7, 8].

## FDA Cancer Drug Approvals in 2025

In the review of the 2025 FDA drug approvals across various therapeutic areas, cancer emerged as the predominant field, followed by cardiology, allergy and inflammation [6]. In total, 46 new drugs were approved in 2025, 16 of which were for the treatment of different oncological and hematological malignancies, accounting for 35% of all the drug approvals. This is in line with the trend observed in preceding years, where cancer is the dominant therapeutic area for drug development, both with regard to biological and small molecular drugs [6]. Table 1 lists the cancer drugs along with their indications, drug classes, orphan drug status, and accelerated approval status [7, 8]. Of the 16 cancer drugs, 15 were classified as new molecular entities, as pembrolizumab for subcutaneous administration is a new formulation of the monoclonal antibody, which was initially approved in 2014 [9]. Pembrolizumab is normally administered as an intravenous infusion; however, in this new formulation, an endoglycosidase (hyaluronidase alfa) has been added to enhance dispersion and permeation in the abdominal subcutaneous tissue, thereby increasing absorption [9].

**Table 1:** Drugs for the treatment of oncological and hematological malignancies approved by the FDA in 2025 [6, 7].

**Drug**	**OD/AA**	**Drug Class**	**Indication**
Datopotamab deruxtecan	No/No	Antibody-drug conjugate	HR-positive, HER2-negative breast cancer
Treosulfan	Yes/No	Chemotherapeutic	Allogeneic hematopoietic stem cell transplantation in patients with AML/MDS
Mirdametinib	Yes/No	Kinase inhibitor	Neurofibromatosis type 1
Vimseltinib	No/No	Kinase inhibitor	Tenosynovial giant cell tumor
Penpulimab	Yes/No	Monoclonal antibody	Nonkeratinizing nasopharyngeal carcinoma
Avutometinib plus defactinib	Yes/Yes	Kinase inhibitors	*KRAS*-mutated low-grade serous ovarian cancer
Telisotuzumab vedotin	No/Yes	Antibody-drug conjugate	NSCLC with high c-Met protein overexpression
Taletrectinib	Yes/No	Kinase inhibitor	*ROS1*-Positive NSCLC
Linvoseltamab	Yes/Yes	Bispecific antibody	Multiple myeloma
Sunvozertinib	No/Yes	Kinase inhibitor	*EGFR* exon 20 insertion mutations in NSCLC
Dordaviprone	Yes/Yes	Small molecule inhibitor	*H3 K27M* mutated glioma
Zongertinib	No/Yes	Kinase inhibitor	*HER2*-mutated non-squamous NSCLC
Pembrolizumab & berahyaluronidase alfa	No/No	Monoclonal antibody plus an endoglycosidase	Multiple (see prescribing information for Keytruda Qlex [9])
Imlunestrant	No/No	Small molecule inhibitor	ER-positive, HER2-negative, *ESR1*-mutated breast cancer
Ziftomenib	Yes/No	Small molecule inhibitor	AML with *NPM1* mutation
Sevabertinib	Yes/Yes	Kinase inhibitor	*HER2*-mutated non-squamous NSCLC

OD, orphan drug; AA, accelerated approval; HR, hormone receptor; AML, acute myeloid leukemia; MDS, myelodysplastic syndrome; NSCLC, non-small cell lung cancer.

Considering the new cancer drugs approved in 2025, kinase inhibitors accounted for nearly half of all approvals. Seven of the 16 drugs (44%) belong to this class, including zongertinib, sevabertinib, taletrectinib, and sunvozertinib, all of which are used to treat different molecular subsets of patients with non-small cell lung cancer (NSCLC). Likewise, two new antibody-drug conjugates were approved in 2025: telisotuzumab vedotin for the treatment of patients with non-squamous NSCLC with high c-Met protein expression and datopotamab deruxtecan for the treatment of HR-positive and HER2-negative breast cancer. Furthermore, it is important to notice that eight of the 16 (50%) approved drugs have received orphan drug designations, indicating that they are for the treatment of rare cancer diseases, as shown in Table 1 [8]. In the US, such a status is designated to drugs or biologics for the treatment of diseases affecting fewer than 200,000 persons or for which the development of the drug will not be profitable within seven years following FDA approval [8]. Additionally, 7 (44%) of the 16 cancer drugs were approved under the FDA Accelerated Approval Program, allowing earlier approval of drugs that treat serious conditions and address unmet medical needs based on a surrogate endpoint [10].

## Efficacy documentation and clinical trial design

Upon reviewing the efficacy documentation for the FDA 2025 approved cancer drugs with respect to clinical trial design, it was found that the majority used the drug–diagnostic co-development model [7]. Ten of the 16 drugs (63%) used an enrichment trial design, in which the study population was enriched with likely responding patients using a drug-specific CDx assay [11]. Table 2 lists the drugs that have employed this design along with their biomarkers and CDx assays. For most drugs listed in Table 2, efficacy has been demonstrated in open-label trials without any comparators. A single-arm design was used for nine of the 16 approved drugs (56%), whereas the remaining seven drugs used a randomized design, which was mostly unblinded. Only vimseltinib for the treatment of tenosynovial giant cell tumors and penpulimab for the treatment of non-keratinizing nasopharyngeal carcinoma have been evaluated in clinical trials using a traditional randomized double-blind design [7].

**Table 2:** **Cancer drugs approved by the FDA in 2025 that utilized the clinical enrichment trial design.** In addition to the generic names of the drugs, the table provides information on biomarkers, specific design characteristics, and available regulatory-approved CDx assays [5, 7].

**Drug**	**CDx Biomarker**	**Trial Design**	**Approved CDx Assay**
Avutometinib plus defactinib	*KRAS*	Single arm	Not available at the time of drug approval
Telisotuzumab vedotin	c-Met	Single arm	Ventana MET (SP44) RxDx Assay
Taletrectinib	*ROS1*	Single arm	Not available at the time of drug approval
Sunvozertinib	*EGFR* exon 20 insertion	Randomized open-label	Oncomine Dx Express Test
Dordaviprone	*H3 K27M*	Single arm	Not available at the time of drug approval
Zongertinib	*HER2*	Single arm	Oncomine Dx Target Test
Pembrolizumab & berahyaluronidase alfa	PD-L1 MSI-H dMMR TMB-H	See prescribing information [9]	PD-L1 IHC 22C3 FoundationOne CDx Ventana MMR RxDx Panel MI Cancer Seek
Imlunestrant	*ESR1*	Randomized open-label	Guardant360 CDx
Ziftomenib	*NPM1*	Single arm	Not available at the time of drug approval
Sevabertinib	*HER2*	Single arm	Oncomine Dx Target Test

**Figure 1:**
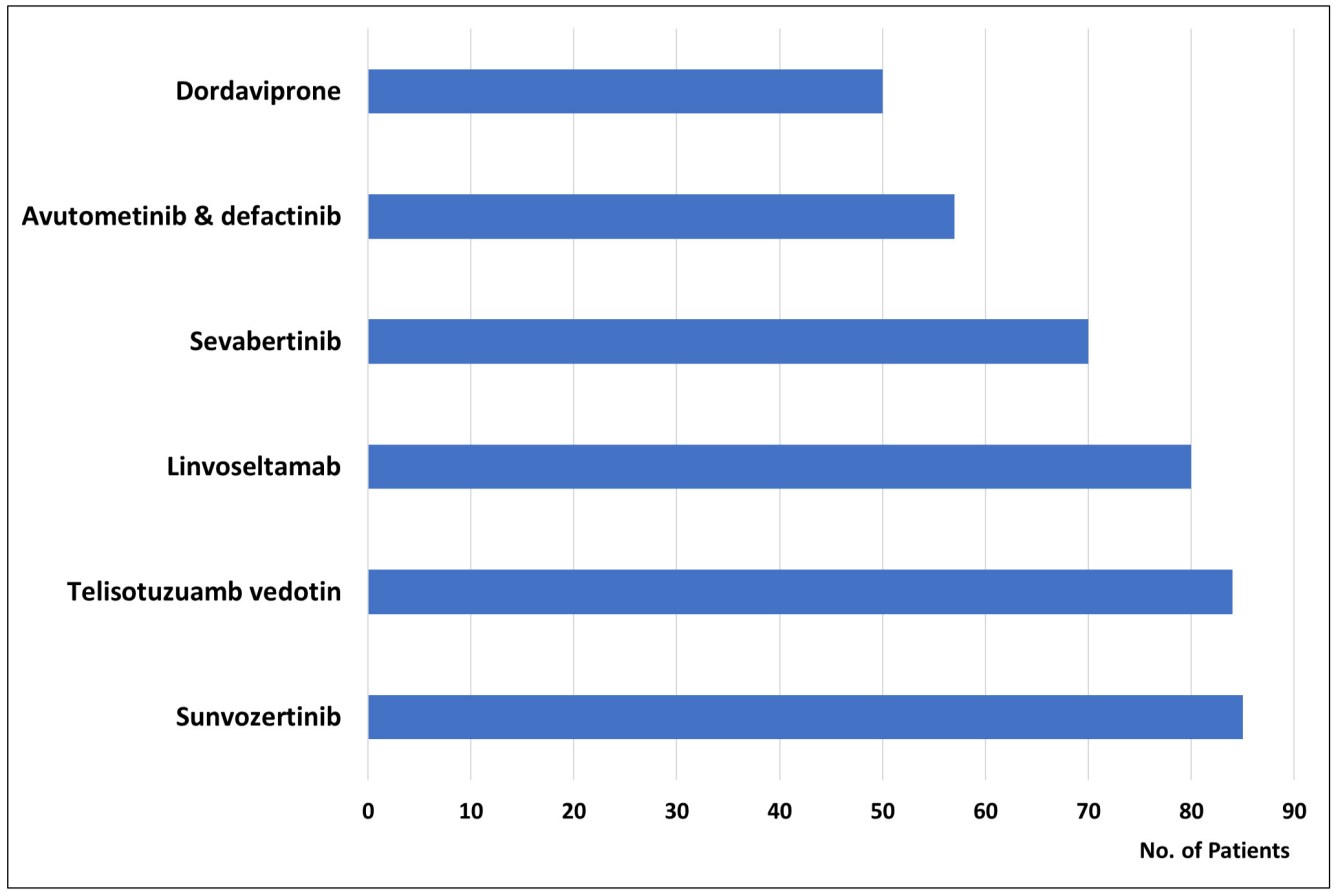
For six of the 16 new cancer drugs approved by the FDA in 2025, efficacy documentation was based on clinical trials that included fewer than 100 patients [7].

The total number of patients included in the clinical development programs to document the efficacy of each of the approved drugs showed considerable variation. The median number of patients per drug was 113, ranging from 57 for avutometinib plus defactinib, indicated for the treatment of KRAS-mutated low-grade serous ovarian cancer, to 732 for datopotamab deruxtecan, indicated for the treatment of HR-positive, HER2-negative breast cancer. As shown in Figure 1, for six of the 16 approved cancer drugs (38%), their efficacy was documented based on clinical trials with a total sample size of less than 100 patients [7]. Among these six drugs, four have received orphan drug designations: dordaviprone, sevabertinib, linvoseltamab, and the combination of avutometinib with defactinib [8]. In general, the number of patients included in clinical development programs for specific drugs tends to be proportional to the size of the target population.

The primary endpoint in the clinical trials that led to the approval of the 16 drugs was, in most cases, based on surrogate endpoints, which were in accordance with the conditions for accelerated approval [10]. The primary endpoint was the objective response rate (ORR) for 12 of the 16 drugs (75 %), often followed by the duration of response (DOR) as a secondary endpoint. In a few randomized studies, progression-free survival (PFS) and overall survival (OS) were used as primary or secondary endpoints [7].

## Companion diagnostic assays

During the clinical development of the drugs listed in Table 1, most employed a drug specific CDx assay for patient enrollment. Table 2 lists these 10 drugs (63%) along with their corresponding predictive biomarkers. Although CDx assays were used during clinical development, they were not always approved concurrently with the drugs in all cases. In fact, this was the situation for six of the 10 drugs that used the clinical enrichment design to document efficacy. Looking at the official FDA full prescribing information for avutometinib plus defactinib, taletrectinib, dordaviprone, and ziftomenib under the section “Patient Selection,” it is stated that an FDA-approved test for the detection of the biomarker in question is not currently available [7].

## Discussion

Currently, cancer is the most prominent therapeutic area in drug development. In 2025, more than one-third of all new drugs approved by the FDA were for the treatment of oncological and hematological malignancies, as in the preceding years [6]. These approvals address a diverse range of indications, from molecular subsets of patients with NSCLC, such as *HER2* mutations, *EGFR* exon 20 insertion, and *ROS1* rearrangements, to rarer conditions, such as neurofibromatosis type 1 and tenosynovial giant cell tumors. Notably, half of the approved drugs received orphan drug designations, indicating an increased focus on meeting unmet needs in small cancer patient populations. The approvals also indicate a strong focus on precision medicine and molecular targeted drug interactions with different signal pathways related to tumor growth, as illustrated by the relatively high number of approved kinase inhibitors, which accounted for 44% of all new cancer drugs in 2025 [7].

A common characteristic of most new approvals was the use of the drug-diagnostic co-development model during clinical development by employing the enrichment trial design. With this design, patients are enrolled in clinical trials based on biomarker status using a drug-specific CDx assay. However, the characteristics of these trials have been debated. Most of them are single-arm trials with a relatively low number of patients, and for six of the 16 cancer drugs approved by the FDA in 2025, efficacy was documented in clinical trials that enrolled fewer than 100 patients [7]. Among these drugs were sevabertinib, dordaviprone, linvoseltamab, and avutometinib plus defactinib, all of which have been designated as orphan drugs and have obtained accelerated approval. For example, in November 2025, the kinase inhibitor sevabertinib was granted accelerated approval for the treatment of locally advanced or metastatic non-squamous NSCLC with *HER2* tyrosine kinase domain-activating mutations. The efficacy of sevabertinib was evaluated in 70 patients from the SOHO-01 study (Cohort D), an open-label, single-arm, multicenter, multicohort clinical trial. The primary endpoints of the trial were the ORR and DOR [12]. For some drugs with relatively limited data supporting their efficacy and safety, it is important to remember that they have been classified as orphan drugs, indicating that they are intended for the treatment of small patient populations. Large randomized clinical trials will be difficult to conduct in these populations, and for some drugs, identifying a relevant comparator will be likewise difficult. Despite the frequent use of the single-arm design and the low number of patients enrolled in these clinical trials, this approach must still be regarded as meaningful, as it has been shown to contribute to the progress of treatment, specifically for several rare cancers. Furthermore, it is important to mention that several of drugs have been approved according to the FDA Accelerated Approval Program. Under this program, early approval can be obtained based on limited data; however, it is mandatory for pharmaceutical and biotech companies to conduct trials to verify the expected clinical benefits of the drug. If subsequent clinical trials fail to demonstrate the anticipated benefits, the FDA may withdraw the drug from the market [10].

For nearly two-thirds of the approved cancer drugs, a CDx assay played an important role during clinical development by enriching the trial populations with likely responding patients. However, when it comes to the concurrent approval of drugs and CDx assays, this seems to fail for a number of drugs. Ten cancer drugs approved by the FDA in 2025 were developed using a clinical enrichment design; however, for four of them, the concurrent regulatory approval of the drugs and CDx assays failed. The unavailability of an analytically and clinically validated CDx assay may pose a challenge to healthcare providers, who may face difficulties in reliably identifying eligible patients for treatment. The CDx assay is an important treatment decision tool that must be available simultaneously with the drug to guide its clinical use and avoid delaying appropriate therapy. In cases where co-approval of a drug and CDx assay is not obtained, the timeframe for the availability of an analytically and clinically validated assay will be included in the drug approval letter as a post-marketing commitment [13]. This was also the case when pembrolizumab was approved in May 2017 for the treatment of adult and pediatric patients with unresectable or metastatic, microsatellite instability-high (MSI-H), or mismatch repair-deficient (dMMR) solid tumors. However, it took nearly five years before FDA-approved assays were available, which for MSI-H was in February 2022 and for dMMR in March of the same year [5]. Such a time lag is not optimal, and pharmaceutical and biotech companies must improve the planning of their drug-diagnostic co-development activities and prioritize CDx development to ensure timely approval. Similarly, regulators must implement stricter coordination and follow-up procedures to avoid such situations in future.

If a CDx assay is essential for the safe and effective use of a new drug, it should be available once the therapeutic agent is approved. Nonetheless, the FDA might choose to approve a new drug without simultaneously approving an assay in cases of serious and life-threatening conditions with no satisfactory alternative treatments [14]. Over the past 5–10 years, we have experienced several instances in which this has been the case [13]. In such situations, patients who are likely to respond to the new drug may be identified using a laboratory-developed test (LDT), provided that such a test is available. The FDA defines an LDT as an in vitro diagnostic test that is manufactured and used in a single laboratory setting [13]. Laboratories that manufacture and use LDT assays are certified under the Clinical Laboratory Improvement Program, which requires analytical validity documentation. However, there are no requirements for clinical validation, which means that for most LDT assays, this type of documentation is missing [13].

## Conclusion

In 2025, the FDA approved a significant number of new cancer drugs, which accounted for 35% of all new drug approvals in the USA. This development was largely driven by targeted therapies, many of which have orphan drug status. Although most of these drugs were developed with CDx assays for patient selection during clinical development, the subsequent concurrent approval of the assay and drug is frequently absent. It is important that an analytical and clinically validated assay is available simultaneously with the drug to enable healthcare providers to correctly identify eligible patients and not delay access to appropriate therapy. Overall, the FDA’s cancer drug approvals for 2025 highlight an ongoing commitment to precision medicine, with several new targeted treatments, such as kinase inhibitors and antibody-drug conjugates, where CDx assays play an important role in identifying the appropriate patient population.

## Abbreviations

CDx: companion diagnostics
dMMR: mismatch repair deficient
DOR: duration of response
IHC: immunohistochemical
LDT: laboratory-developed tests
MSI-H: microsatellite instability-high
NSCLC: non-small cell lung cancer
ORR: objective response rate
OS: overall survival

## Declarations

### Availability of data and materials

The following data sources were used: Mullard A. 2025 FDA approvals. Nat Rev Drug Discov. 2026;25:81–7. [DOI: 10.1038/d41573-026-00001-z]; Drugs@FDA: FDA-approved drugs. Available from: https://www.accessdata.fda.gov/scripts/cder/daf/; Search Orphan Drug Designations and Approvals. Available from: https://www.accessdata.fda.gov/scripts/opdlisting/oopd/; List of Cleared or Approved Companion Diagnostic Devices (In Vitro and Imaging Tools). Available from: https://www.fda.gov/medical-devices/in-vitro-diagnostics/list-cleared-or-approved-companion-diagnostic-devices-in-vitro-and-imaging-tools.

### Copyright

© The Author(s) 2026.
